# The Diagnostic Potential and Regulatory Mechanisms of miR-3180-3p in Metabolic Dysfunction–Associated Fatty Liver Disease with Respect to Inflammation and Oxidative Stress

**DOI:** 10.5152/tjg.2025.25346

**Published:** 2025-11-21

**Authors:** Kexiu Song, Peng Qi

**Affiliations:** 1 Department of Endocrinology, Affiliated Hospital of Qingdao University, Qingdao, People’s Republic of China; 2 Department of Gastroenterology, Affiliated Hospital of Qingdao University, Qingdao, People’s Republic of China

**Keywords:** Diagnostic biomarker, inflammatory oxidative stress, metabolic dysfunction–associated steatotic liver disease, miR-3180-3p, SKI

## Abstract

**Background/Aims:**

miR-3180-3p has been linked to hepatocellular carcinoma progression, but its role and molecular mechanisms in metabolic dysfunction-associated steatotic liver disease (MASLD) are still unclear. The objective is to explore whether miR-3180-3p serves as a diagnostic biomarker for MASLD and elucidate its function in mediating hepatic inflammatory and oxidative stress responses.

**Materials and Methods:**

This cross-sectional study included 117 patients with MASLD and 100 healthy controls. Serum and cellular RNA were extracted for real-time polymerase chain reaction analysis. In vitro experiments utilized HepG2 cells treated with free fatty acids (FFAs) to mimic MASLD-related stress conditions, evaluating miR-3180-3p expression levels and its effects on oxidative stress markers (malondialdehyde, superoxide dismutase) and inflammatory cytokines (tumor necrosis factor-α, interleukin-6 (IL-6), IL-1β). Target genes of miR-3180-3p were identified using bioinformatics and dual-luciferase assays.

**Results:**

Serum miR-3180-3p levels were significantly higher in MASLD patients than controls, showing strong diagnostic value and potential as a biomarker. Multivariate analysis identified it, along with C-reactive protein (CRP) and high-density lipoprotein (HDL)-cholesterol, as an independent risk factor for MASLD. Its expression positively correlated with triglycerides, low-density lipoprotein-cholesterol, white blood cell count, CRP, Fibrosis-4, and oxidative stress markers (OA, PA), but negatively with HDL cholesterol. In FFA-treated cells, higher miR-3180-3p was linked to increased oxidative stress and inflammation. *SKI*, a direct target of miR-3180-3p, when silenced, reversed the protective effects of miR-3180-3p inhibition against FFA-induced cellular damage.

**Conclusion:**

miR-3180-3p is a promising diagnostic biomarker for MASLD and contributes to disease progression by enhancing hepatic inflammation and oxidative stress via targeting *SKI*.

**Main Points**

- miR-3180-3p may serve as a potential diagnostic biomarker for metabolic dysfunction–associated steatotic liver disease (MASLD).
- miR-3180-3p is independently correlated with the severity of MASLD.
- Inhibition of miR-3180-3p may alleviate oxidative stress in hepatocytes.
- Inhibition of miR-3180-3p may alleviate the inflammatory response in hepatocytes.
- miR-3180-3p targets *SKI* to regulate the progression of MASLD.

## Introduction

Metabolic dysfunction–associated steatotic liver disease (MASLD) is the most common chronic liver condition globally, posing a substantial public health challenge.^1^ Previously, MASLD was referred to as non-alcoholic fatty liver disease (NAFLD) in June 2023, a multi-society Delphi consensus statement on a new fatty liver disease nomenclature was published, introducing the term MASLD and effectively retiring the term NAFLD.^2^ Metabolic dysfunction–associated steatotic liver disease has the potential to advance from non-alcoholic fatty liver (NAFL) to metabolic dysfunction–associated steatohepatitis (MASH), and in more severe cases, can lead to cirrhosis or hepatocellular carcinoma (HCC), thereby posing a substantial threat to human health.^3^^-^^5^ The etiology of MASLD is complex and involves multiple factors.^6^ At its core, the pathogenesis is driven by an imbalance in hepatic lipid metabolism mediated by insulin resistance.^7^^,^^8^ This is manifested by increased hepatic triglyceride synthesis, reduced fatty acid oxidation, and impaired very low-density lipoprotein (LDL) lipid secretion, ultimately resulting in intrahepatic fat accumulation.^9^^,^^10^ Obesity plays a critical role, with free fatty acids (FFAs) released from visceral abdominal fat being directly transported to the liver, exceeding the metabolic capacity of hepatocytes.^11^ Additionally, adipose tissue secretes inflammatory cytokines, including tumor necrosis factor-α (TNF-α) and interleukin-6 (IL-6), which trigger endoplasmic reticulum stress and oxidative stress in liver cells, exacerbating fat deposition and inflammatory damage.^12^ Metabolic disorders, such as abnormalities in glucose and lipid metabolism, metabolic syndrome, and polycystic ovary syndrome, further drive disease progression by promoting insulin resistance and dysregulation of lipid metabolism.^13^ In terms of dietary habits, a high-calorie, high-fat diet increases fatty acid supply to the liver or induces lipid peroxidation, thereby aggravating fat accumulation. Physical inactivity reduces glucose uptake by skeletal muscles, exacerbating systemic insulin resistance and impairing mitochondrial function, which further disrupts the hepatic energy metabolic balance.^14^

Currently, liver biopsy remains the gold standard for clinically diagnosing MASLD.^15^ However, it is associated with risks of biopsy-related complications and diagnostic bias due to sampling errors. MicroRNA (miRNA) is a class of non-coding small RNA molecules (approximately 19-25 nucleotides in length) transcribed by RNA polymerase II. It regulates gene expression by targeting mRNAs and plays a crucial role in the core pathological mechanisms of MASLD, including metabolic disorders, inflammatory responses, and oxidative stress.^16^ As a promising biomarker, the aberrant expression profile of miRNAs not only reflects pathological changes in the liver microenvironment but also captures the dynamic alterations in this microenvironment across different disease stages.^17^ These dynamic changes further provide novel targets for early diagnosis, prognostic evaluation, and personalized intervention strategies. A previous study demonstrated that miR-3180-3p contributes to palmitate-induced insulin resistance by inhibiting insulin-mediated IRS-1 phosphorylation.^18^ Notably, overexpression of miR-3180-3p suppresses HCC cell proliferation, migration, and invasion, while reversing the oncogenic effects of SNHG17.^19^ This suggests that miR-3180-3p may serve as a regulatory factor in the progression from MASLD to HCC. Nevertheless, the diagnostic efficacy of miR-3180-3p in MASLD and its molecular mechanisms underlying the activation of hepatic inflammatory cascades and oxidative stress-induced injury require further investigation through high-quality clinical studies and basic experiments.

Based on the aforementioned background, this study aimed to comprehensively assess the effectiveness of miR-3180-3p as a diagnostic biomarker by examining its differential expression in the serum of healthy individuals and those with MASLD. This study aims to clarify the potential mechanism by which miR-3180-3p/*SKI* influences inflammation and oxidative stress during the progression of NAFLD, thereby providing experimental evidence for the development of miRNA-based, non-invasive diagnostic systems and intervention strategies that target key nodes of liver injury.

## Materials and Methods

### Clinical Data Collection

The present study enrolled 117 patients diagnosed with MASLD based on pathological examination of liver biopsy specimens between April 2021 and September 2023. Additionally, 100 individuals with normal liver function, identified through routine physical examinations at the same hospital during the same period, were selected as controls after detailed evaluations confirming the absence of liver diseases. The research received approval from the Ethics Committee of The Affiliated Hospital of Qingdao University Affiliated Hospital of Qingdao University (Date: April 14, 2021; Approval No: 20210105) and followed the ethical principles set forth in the 1975 Declaration of Helsinki. Before sample collection, all participants were thoroughly informed about the study and provided written consent.

Blood samples were collected via venipuncture following an overnight fast of 8-12 hours. Whole blood was spun at 3000 revolutions per minute for 10 minutes to isolate the serum, which was then stored at −80°C until further analysis. Clinical data, including age, gender, body mass index (BMI), and laboratory results such as total cholesterol (TC), triglycerides (TG), white blood cell count (WBC), aspartate aminotransferase (AST), diabetes mellitus, and essential hypertension, were recorded. Based on previous studies, the Fibrosis-4 (FIB-4) index was calculated for each patient.^20^

### Serum and Cell Total RNA Extraction

After the frozen serum samples were thawed on ice, total serum RNA was extracted using MolPure® Serum/Plasma miRNA Kit (9332ES50, Yeasen) following the manufacturer’s protocol. Total cellular RNA was extracted with Cell/Tissue miRNA Kit (19331ES50, Yeasen). All RNA samples extracted were promptly frozen in liquid nitrogen and stored at −80°C to prevent RNA degradation and ensure the stability of follow-up experiments.

### Real-time Fluorescence Quantitative Polymerase Chain Reaction

RNA was converted into cDNA through reverse transcription using the miRNA first strand cDNA Synthesis Kit (stem-loop method) (MR101, Vazyme, China). The expression levels of miR-3180-3p in both serum and cells were measured via real-time fluorescence-based quantitative polymerase chain reaction (qPCR), employing the miRNA Unimodal SYBR qPCR Master Mix (MQ102, Vazyme, China). *HMBS* was used as the internal control for normalization.

cDNA was synthesized from RNA using the HiScript III RT SuperMix for qPCR (+gDNA wiper, R323-01, Vazyme, China). The expression levels of *SKI*, *ZNF385A*, and *MIDN* in cells were quantified using ChamQ Universal SYBR qPCR Master Mix (Q711-02, Vazyme, China), with *GAPDH* serving as the internal reference.

### Cell Culture

The human HCC cell line HepG2, acquired from the Shanghai Cell Bank (Shanghai, China), was cultured in DMEM medium containing 10% fetal bovine serum, 1% L-glutamine, and 1% penicillin-streptomycin solution. The cells were maintained in a humidified incubator at 37°C with 5% CO_2_ and subcultured every 2–3 days.

### Cell transfection and treatment

HepG2 cells were seeded in culture dishes and transfected when they reached 70%-80% confluence. The miR-3180-3p inhibitor, si-SKI and its negative control (50 nM, RiboBio) were introduced into the cells following the Lipofectamine 3000 (Invitrogen) reagent protocol. After transfection, the cells were cultured at 37°C with 5% CO_2_ for 24 hours. Following this, the cells were exposed to a fatty acid mixture of palmitic acid (PA) and oleic acid (OA) at a molar ratio of 1 : 2. After an additional 24-hour incubation, the cells were harvested using trypsinization, collected by centrifugation, rapidly frozen in liquid nitrogen, and stored at −80°C for subsequent analysis.

### Cellular Inflammation and Oxidative Stress Detection

Levels of inflammatory cytokines (TNF-α, IL-6, and IL-1β) in cell cultures were quantified using an ELISA kit (TransGen Biotech) following the manufacturer’s instructions.

The MDA levels in cells were quantified using the Lipid Peroxidation MDA Assay Kit (S0131S, Beyotime) following the manufacturer’s protocol. Superoxide dismutase (SOD) activity in cells was measured using the Total Superoxide Dismutase Assay Kit with WST-8 (S0101S, Beyotime).

### MicroRNA Target Prediction

The target genes of miR-3180-3p were predicted using the miRTarBase (https://mirtarbase.cuhk.edu.cn/~miRTarBase/miRTarBase_2025/php/index.php), TargetScan (https://www.targetscan.org/vert_72/), miRDB (https://mirdb.org/mirdb/index.html), and starBase (https://rnasysu.com/encori/agoClipRNA.php?source=mRNA) databases. A Venn analysis was performed to identify the overlapping targets among these 4 databases. The TargetScan database was further used to predict the binding sites between miR-3180-3p and its target genes.

### Dual-luciferase Assay

Plasmids containing the wild-type (SKI-WT) and mutant (SKI-MUT) 3’ UTR sequences of *SKI* were constructed. These plasmids were subsequently co-transfected with miR-3180-3p mimics, inhibitors, or negative control sequences into cultured cells. Luciferase activity was measured 48 hours post-transfection following the manufacturer’s protocol for the Dual-Luciferase Reporter Assay Kit (11402ES60, Yeasen). The direct interaction between miR-3180-3p and the SKI 3’ UTR was evaluated by comparing luciferase activity across the different transfection groups.

### Statistical Analysis

Statistical analyses were performed using SPSS 26.0 software (IBM SPSS Corp.; Armonk, NY, USA), while data visualization was achieved through GraphPad Prism 10 (GraphPad Software; San Diego, USA). The diagnostic performance of miR-3180-3p in MASLD patients was evaluated using receiver operating characteristic (ROC) curves, with the area under the curve (AUC) and its corresponding 95% CI being estimated. To identify independent risk factors linked to the severe progression of MASLD, binary logistic regression analysis was utilized, and the odds ratio (OR) along with the 95% CI were determined. Pearson correlation analysis was performed to evaluate the association between miR-3180-3p expression levels and disease severity in patients, where the correlation coefficient (r) was calculated and tested for statistical significance. All in vitro experiments were independently replicated 3 times, and the findings are expressed as mean ± standard error of the mean (SEM). For statistical comparisons, 2-tailed *t*-tests were applied for pairwise comparisons between 2 groups, 1/2o-way ANOVA with Tukey’s post hoc tests for comparisons involving multiple groups, and repeated-measures ANOVA for analyzing data across different time points between 2 groups. A *P* value less than .05 was deemed statistically significant.

## Results

miR-3180-3p could potentially act as a diagnostic biomarker for individuals with MASLD.

After analyzing and comparing the general information and biochemical indices of the subjects, no significant differences were observed between the 2 groups with respect to age, gender, BMI, TC, hypertension, or diabetes (*P >* .05, Table 1). However, TG, LDL-C, WBC, C-reactive protein (CRP), AST, and alanine aminotransferase levels were markedly increased in the MASLD group compared to the normal group (*P* < .001). Conversely, the MASLD group exhibited significantly reduced high-density lipoprotein (HDL-C) levels compared to the normal group (*P* = .002).

**Figure 1.**
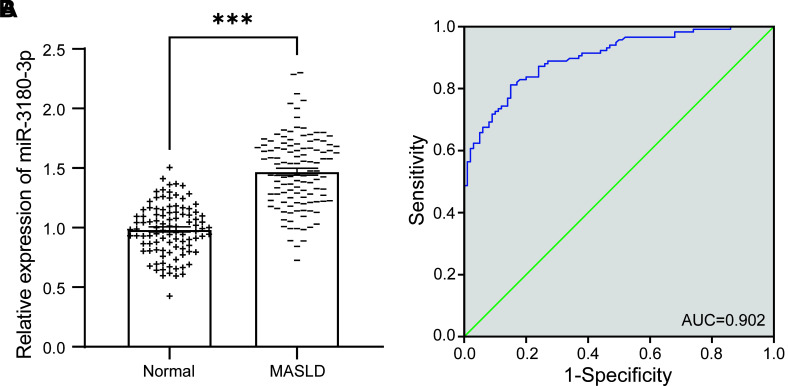
ROC analysis was performed to assess the expression difference and diagnostic value of serum miR-3180-3p in MASLD patients relative to the healthy population. (A) Comparison of serum miR-3180-3p expression between MASLD patients and normal individuals; (B) ROC analysis of the diagnostic value of miR-3180-3p, ***P < .001.

By comparing the levels of miR-3180-3p in the serum of the normal group and the MASLD group, it indicated that miR-3180-3p was significantly elevated in the MASLD group, demonstrating a notable difference when compared to the normal group (*P* < .001, Figure 1A). Furthermore, ROC analysis demonstrated that miR-3180-3p exhibited significant diagnostic potential for MASLD, with an AUC of 0.902 (95% CI: 0.863-0.941, *P* < .0001) (Figure 1B).

miR-3180-3p, CRP, WBC, TG, and LDL were identified as significant risk factors for MASLD diagnosis.

**Figure 2.**
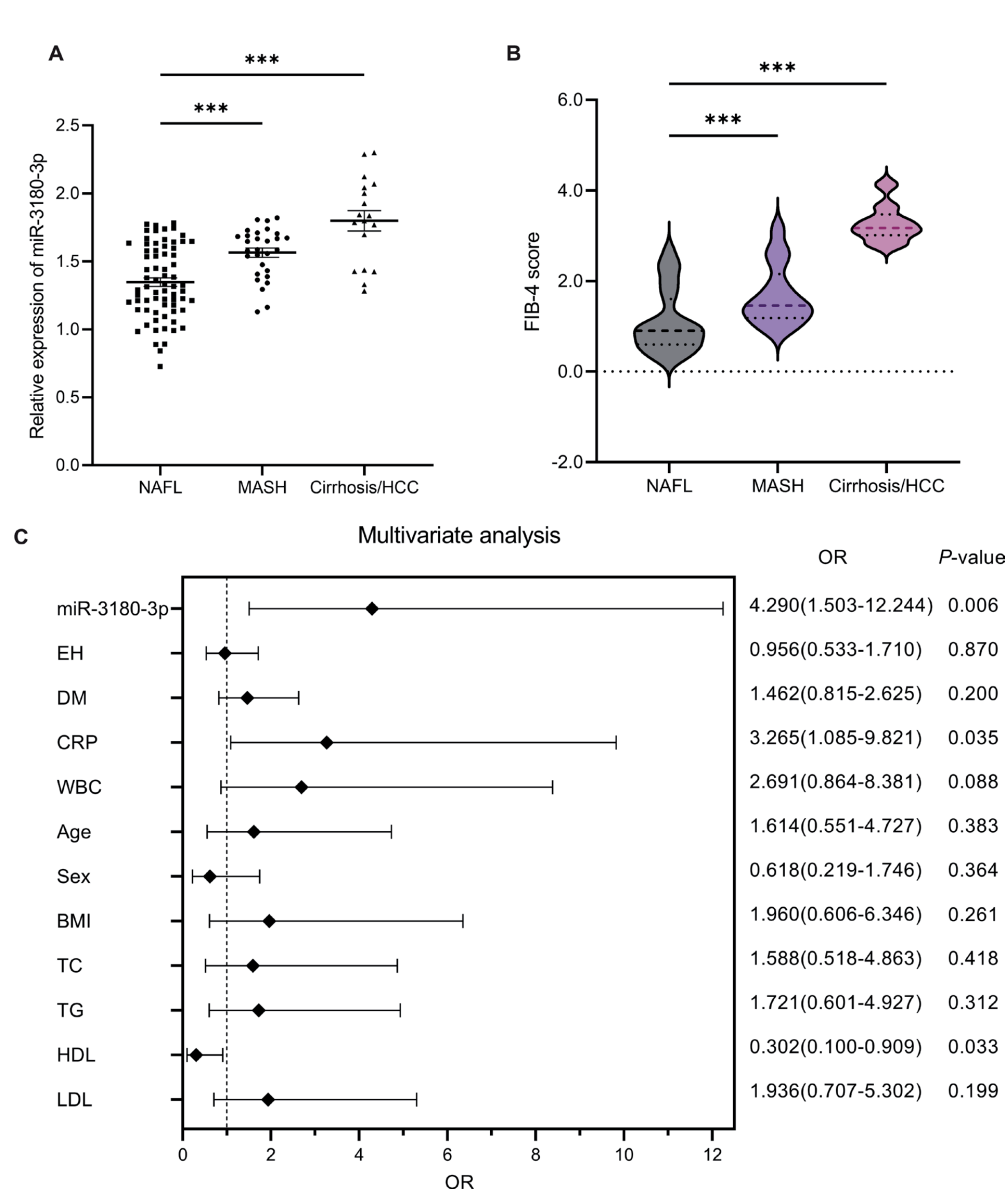
A logistic regression analysis was conducted to investigate the association between serum miR-3180-3p expression levels and risk factors related to disease progression in MASLD patients with different stages of severity. (A) The expression levels of miR-3180-3p in the serum of patients with varying degrees of disease severity; (B) The distribution of FIB-4 scores among patients with varying severity levels; (C) A logistic regression analysis was performed to identify the risk factors associated with severe disease progression in patients with MASLD, ***P < .001.

Based on clinical diagnoses, patients with fatty liver disease were categorized into 3 groups: NAFL, MASH, and MASH-related cirrhosis or HCC. The serum levels of miR-3180-3p were measured, revealing significant upregulation of miR-3180-3p in individuals with MASH, as well as in those with MASH-related cirrhosis or HCC, compared to individuals with NAFL (*P* < .001, Figure 2A). Through calculation of the FIB-4 index across the 3 patient groups, it was observed that the FIB-4 index increased progressively with the severity of the disease (*P* < .001, Figure 2B).

In order to enhance the diagnostic capability of miR-3180-3p for MASLD in the general population, a multifactorial logistic regression analysis was employed to construct a diagnostic model by integrating miR-3180-3p with other clinical indices. Multivariate logistic regression analysis indicated that miR-3180-3p (OR = 4.290, 95% CI: 1.503-12.244, *P* = 0.006), CRP (OR = 3.265, 95% CI: 1.085-9.821, *P* = .035), and HDL-C (OR = 0.302, 95% CI: 0.100-0.909, *P* = .033) were independent risk factors for severe progression in patients with MASLD (Figure 2C).

### Correlation Analysis Between miR-3180-3p Expression Levels and Clinical Biochemical Indicators

The results of Pearson’s correlation analysis indicated that the expression level of miR-3180-3p showed a significant association with multiple clinical biochemical markers (*P* < .05, Table 2). Specifically, miR-3180-3p showed a substantial positive association with TG (*r* = .576), LDL-C (*r* = 0.539), WBC (*r* = 0.557), CRP (*r* = 0.642), OA (*r* = 0.533), PA (*r* = 0.717), and FIB-4 (*r* = 0.739). Notably, miR-3180-3p showed a significant negative association with HDL-C (*r* = −0.619).

The inhibition of miR-3180-3p mitigates fatty acid-induced inflammatory and oxidative stress responses in liver cells.

**Figure 3.**
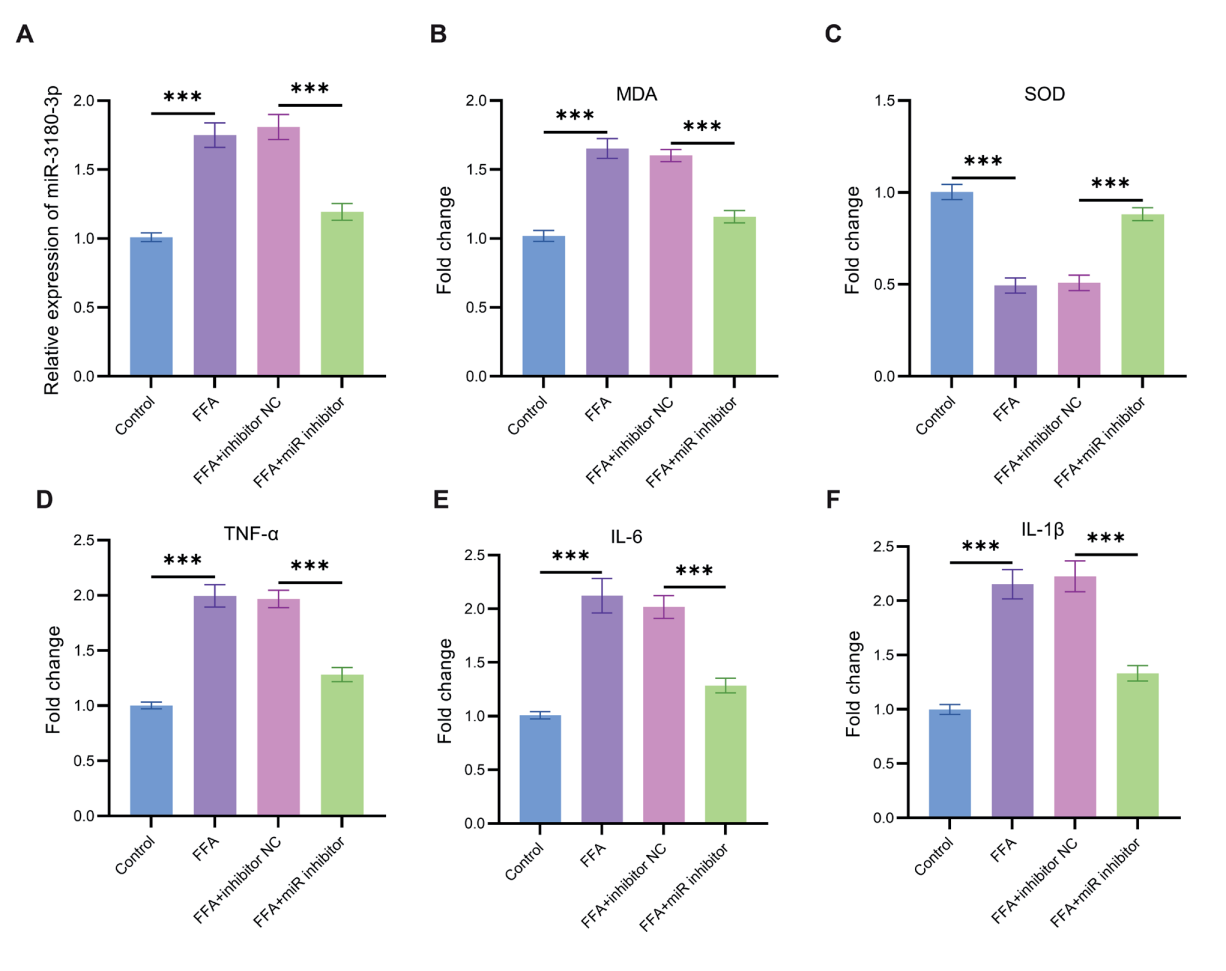
Examination of miR-3180-3p expression levels and alterations in oxidative stress markers and inflammatory factors in HepG2 cells exposed to fatty acid treatment. (A) The expression levels of miR-3180-3p in HepG2 cells treated with a mixture of fatty acids were examined; (B) Changes in the levels of oxidative stress markers MDA; (C) Changes in the levels of oxidative stress markers SOD; (D) Expression levels of inflammatory factors TNF-α; (E) Expression levels of inflammatory factors IL-6; (F) Expression levels of inflammatory factors IL-1β, ***P < .001.

In order to investigate the role of miR-3180-3p in MASLD more thoroughly, this study examined the expression of miR-3180-3p under treatment with a fatty acid mixture (FFA; OA: PA = 2 : 1) (Figure 3A). In comparison to the normal control group, FFA treatment led to a significant upregulation in the expression of miR-3180-3p. After FFA treatment, transfection with a miR-3180-3p inhibitor led to a substantial decrease in miR-3180-3p expression, with levels significantly lower than those in the FFA-treated group (*P* < .05).

In the assessment of oxidative stress markers, FFA treatment markedly increased the level of lipid peroxidation product malondialdehyde (MDA) (Figure 3B) and significantly decreased the activity of the antioxidant enzyme SOD (Figure 3C), indicating enhanced oxidative damage. Transfection with the miR-3180-3p inhibitor led to a decrease in MDA levels and an increase in SOD activity (*P* < .05).

Further investigation into the regulatory effects of miR-3180-3p on inflammatory factors revealed that treatment with FFA resulted in a significant increase in the expression levels of TNF-α (Figure 3D), IL-6 (Figure 3E), and IL-1β (Figure 3F). After transfection with the miR-3180-3p inhibitor, the concentrations of TNF-α, IL-6, and IL-1β showed a significant decrease when compared with the FFA-treated group (*P* < .05).

*SKI* was identified as a target gene of miR-3180-3p.

**Figure 4.**
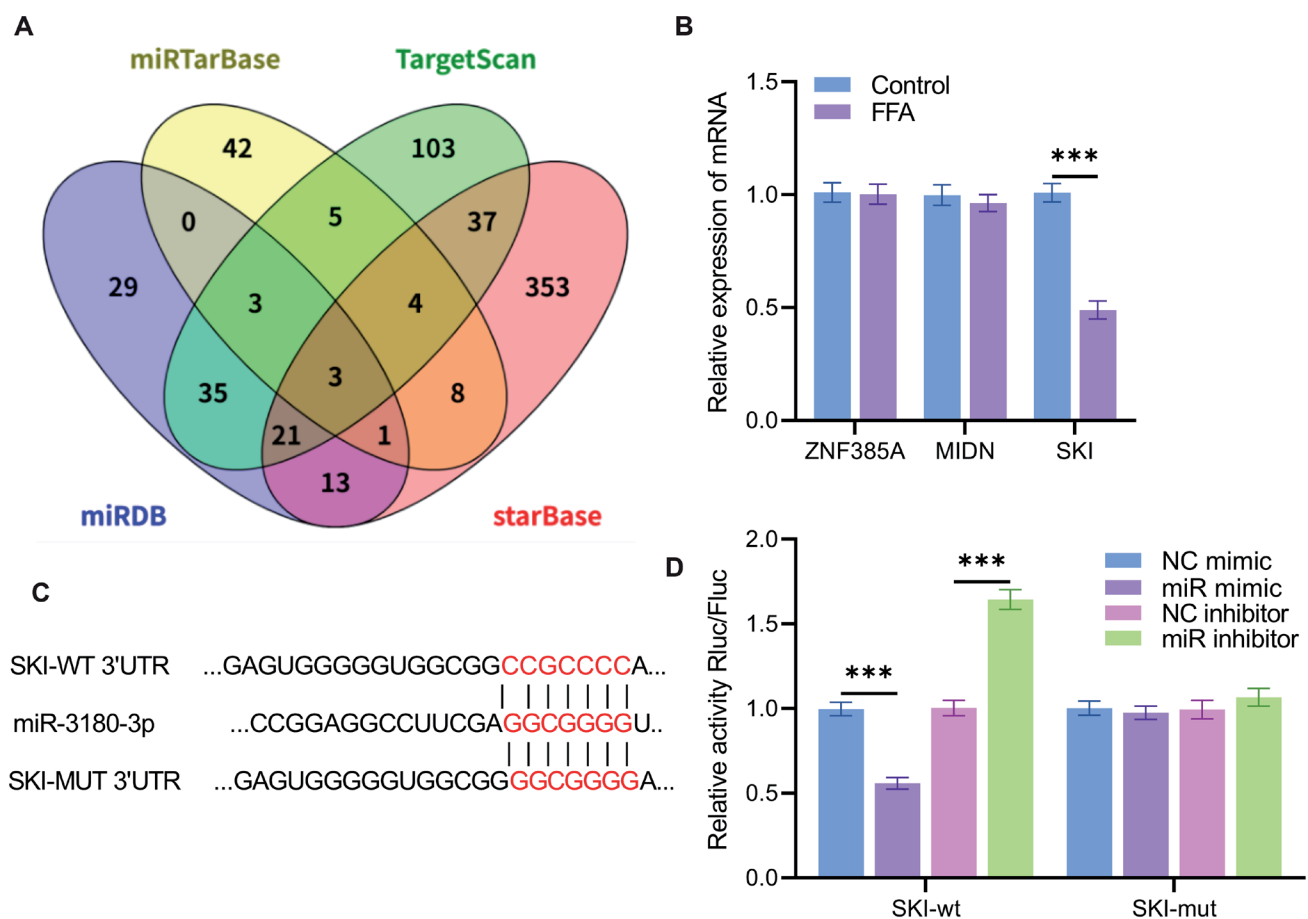
SKI has been identified as a target gene of miR-3180-3p. (A) A Venn diagram illustrates the overlapping target genes of miR-3180-3p predicted by the miRTarBase, TargetScan, miRDB, and starBase databases; (B) The relative mRNA expression levels of ZNF385A, MIDN, and SKI following FFA treatment of cells; (C) The binding site between miR-3180-3p and SKI; (D) The dual-luciferase reporter assay confirmed the direct binding interaction between miR-3180-3p and SKI, ***P < .001.

Through Venn analysis of the predicted target genes in the databases, 3 overlapping genes were identified: *ZNF385A*, *MIDN*, and *SKI* (Figure 4A). The qPCR results demonstrated that SKI expression was significantly downregulated in cells treated with FFA (*P* < .001, Figure 4B). TargetScan prediction indicated that miR-3180-3p has potential binding sites within the SKI 3’UTR (Figure 4C).

Dual-luciferase reporter assays revealed that in the *SKI-WT* group, luciferase activity was significantly reduced following transfection with miR-3180-3p mimics and significantly increased after transfection with miR-3180-3p inhibitors (*P* < .001, Figure 4D). In contrast, no significant changes in luciferase activity were observed in the *SKI-MUT* group under the same treatment conditions(*P >* .05).

*SKI* participates in the regulation of oxidative stress and inflammatory responses in FFA-treated cells.

**Figure 5.**
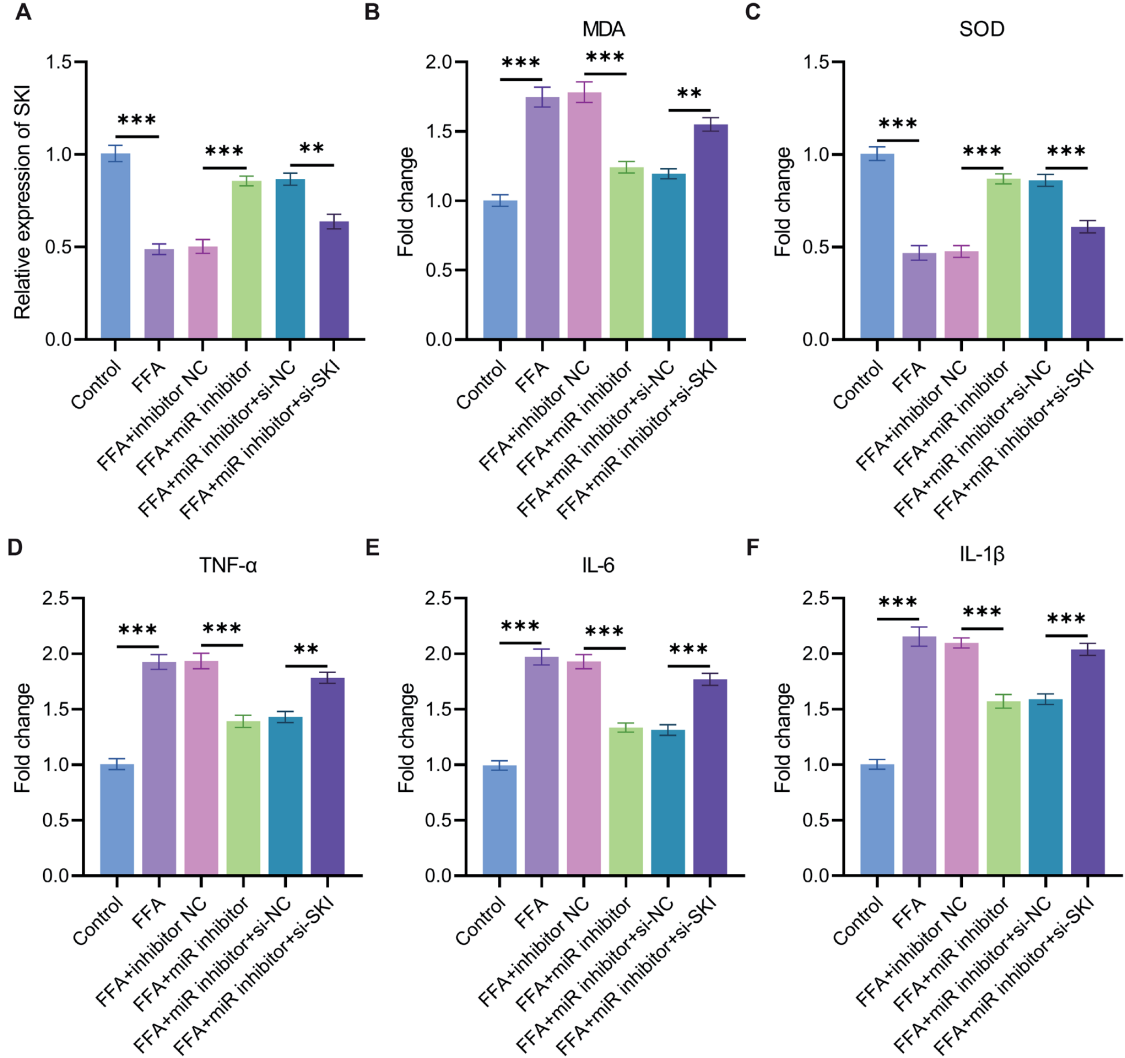
Functional validation of SKI’s role in regulating oxidative stress and inflammation during FFA-induced cell injury. (A) The relative expression level of SKI across different treatment groups was determined by qPCR; (B) Changes in the levels of oxidative stress markers MDA; (C) Changes in the levels of oxidative stress markers SOD; (D) Expression levels of inflammatory factors TNF-α; (E) Expression levels of inflammatory factors IL-6; (F) Expression levels of inflammatory factors IL-1β, **P < .01, ***P < .001.

Compared with the control group, the relative expression level of *SKI* was significantly downregulated in cells treated with FFA. Transfection of the miR-3180-3p inhibitor partially restored *SKI* expression, whereas co-transfection of the miR-3180-3p inhibitor and si-*SKI* significantly reduced *SKI* expression (*P* < .001, Figure 5A).

Free fatty acid treatment significantly increased the MDA level in cells. Transfection of the miR-3180-3p inhibitor reduced the MDA level, whereas co-transfection of the miR-3180-3p inhibitor and si-*SKI* significantly elevated it (*P* < .001, Figure 5B).

Free fatty acid treatment significantly reduced the SOD level in cells. Transfection with the miR-3180-3p inhibitor increased the SOD level, whereas co-transfection with the miR-3180-3p inhibitor and si-*SKI* significantly decreased it (*P* < .001, Figure 5C).

Free fatty acid treatment significantly increased the levels of TNF-α, IL-6, and IL-1β in cells. Transfection of the miR-3180-3p inhibitor reduced these cytokine levels, whereas co-transfection of the miR-3180-3p inhibitor and si-*SKI* significantly elevated them (*P* < .001, Figure 5D-F).

## Discussion

Research indicates that miRNAs, which regulate multiple complementary mRNA targets at the post-transcriptional level, exhibit substantial prognostic and predictive value in pathological liver diseases, including MASLD.^21^ Prior research has demonstrated that miR-34a expression in the liver is increased in MASLD patients and continues to rise as the disease advances.^22^^,^^23^ Hepatic miR-122 levels rise during the early stages of MASLD but gradually decrease as the condition progresses to MASH and fibrosis.^24^ In this study, miR-3180-3p was found to be significantly upregulated in the serum of MASLD patients and demonstrated high diagnostic accuracy, as evidenced by ROC curve analysis with an AUC of 0.902. This suggests that miR-3180-3p can effectively differentiate MASLD patients from healthy controls. As a non-invasive biomarker, miR-3180-3p is expected to play a pivotal role in clinical diagnosis. Changes in its expression may reflect pathological alterations in the liver microenvironment of MASLD patients, offering new insights into the early detection and monitoring of the disease.

Metabolic dysfunction–associated steatotic liver disease may progress to MASH, cirrhosis, and HCC, a pathological process closely associated with metabolic disorders and chronic inflammation.^5^ Earlier studies have shown that miR-3180-3p is linked to insulin resistance in hepatocytes and contributes to the regulation of HCC progression.^18^^,^^19^ This study further observed that in MASLD patients, serum levels of miR-3180-3p increased with disease severity and were significantly upregulated, particularly in MASH and cirrhosis/HCC subgroups, and show a strong positive correlation with the FIB-4 index, a well-established marker of liver fibrosis.^25^ Furthermore, logistic regression analysis identified miR-3180-3p, CRP, and HDL-C as significant predictors of the advancement of MASLD severity. These findings provide a novel perspective for early clinical screening: miR-3180-3p can be used in conjunction with traditional metabolic-inflammatory markers (CRP and HDL-C) to enhance diagnostic accuracy. CRP promotes the progression of MASLD to MASH by mediating the insulin signaling pathway, mitochondrial metabolic abnormalities, and the NF-κB inflammatory pathway, while the anti-inflammatory properties of HDL-C make it a potential marker for evaluating MASLD and liver fibrosis.^26^^,^^27^

This study demonstrated that aberrant expression of miR-3180-3p acts as a pivotal molecular node driving the progression of MASLD to liver fibrosis, cirrhosis, and even HCC by mediating metabolic disorders, chronic inflammation, and oxidative stress injury.^28^ At the lipid metabolism level, miR-3180-3p expression was significantly positively correlated with serum TG and low-density lipoprotein cholesterol (LDL-C)—core markers of insulin resistance that trigger lipotoxic effects by promoting the buildup of FFA in hepatocytes.^29^ The inverse correlation between HDL-C and miR-3180-3p suggests that miR-3180-3p may antagonize HDL-C-mediated reverse cholesterol transport and anti-inflammatory functions, exacerbating intrahepatic lipid deposition and insulin resistance, thereby laying the metabolic foundation for the transformation of MASLD to MASH.^30^ Regarding inflammatory mechanisms, the positive correlations between miR-3180-3p and WBC and CRP confirmed its role in synergistically activating the NF-κB pathway.^26^ Treatment of hepatocytes with FFA upregulated miR-3180-3p, which was associated with a 62% increase in TNF-α, IL-6, and other pro-inflammatory factors.^31^ These factors intensified the inflammatory process, leading to the activation of hepatic stellate cells and facilitating the development of liver fibrosis.^32^ At the oxidative stress level, the strong positive correlation between miR-3180-3p and OA and PA was consistent with its functions of promoting lipid peroxidation (MDA) and inhibiting antioxidant enzymes (SOD). Inhibition of miR-3180-3p significantly reversed these injuries, suggesting that miR-3180-3p exacerbates oxidative damage, accelerating apoptosis and DNA damage in hepatocytes and providing a molecular trigger for HCC.^33^ Notably, miR-3180-3p levels were significantly higher in the MASH and cirrhosis/HCC subgroups than in the NAFL group, further confirming the positive correlation between miR-3180-3p expression intensity and the pathological grade of MASLD. miR-3180-3p constitutes the core signaling axis driving the pathological escalation of MASLD through the triangular mechanism of “metabolic disorder - inflammatory activation - oxidative stress.” Its dual value as a diagnostic marker and therapeutic target provides a novel perspective for disease stratification and intervention.

*SKI*, a transcriptional co-repressor, has been implicated in suppressing excessive inflammatory responses and maintaining cellular redox homeostasis in various tissues.^34^^,^^35^ *SKI* plays an important regulatory role in a variety of pathophysiological processes, including liver regeneration, vascular smooth muscle regeneration, and wound healing.^36^ In recent years, numerous studies have demonstrated that *SKI* plays a role in the progression of liver fibrosis and cirrhosis.^37^^,^^38^ In this study, *SKI* was identified as the target gene of miR-3180-3p based on bioinformatics prediction and dual-luciferase assay results. *SKI* expression was downregulated in FFA-treated cells, and miR-3180-3p inhibition restored *SKI* levels, which in turn alleviated oxidative stress and inflammation. Conversely, silencing *SKI* reversed the protective effects of miR-3180-3p inhibition, confirming that *SKI* mediates the regulatory effects of miR-3180-3p. This mechanism is consistent with the regulation of MASLD progression by other miRNAs via their target genes.^39^ Together, these findings establish the miR-3180-3p/SKI axis as a novel molecular pathway driving MASLD pathogenesis, offering a potential therapeutic target for mitigating disease progression.

The current research has a few limitations that need to be recognized. Firstly, the comparatively limited sample size may result in biased estimation of effect sizes, necessitating further validation with larger cohorts. Second, only the HepG2 cell line was utilized for mechanistic investigations, which exhibits metabolic characteristics distinct from those of primary stem cells or hepatocytes. Therefore, the miR-3180-3p upregulation and inflammatory factor release observed in FFA treatment experiments might not fully represent the complex interactions among hepatocytes, stellate cells, and immune cells in vivo. Future work will employ primary hepatocytes and liver organoid models to simulate the complex intercellular interactions within the liver microenvironment during the progression of NAFLD and to further validate the functional role of miR-3180-3p.

In summary, this study demonstrates that miR-3180-3p is significantly upregulated in the serum of patients with MASLD and exhibits high diagnostic accuracy, making it a promising non-invasive biomarker for the disease. Its expression positively correlates with disease severity, as evidenced by strong associations with TG, LDL-cholesterol, CRP, FIB-4, and markers of oxidative stress (OA, PA), while showing an inverse correlation with HDL-cholesterol, further supporting its potential as an indicator of disease progression. miR-3180-3p promotes hepatic inflammation and oxidative stress in FFA-induced hepatocytes by directly targeting and downregulating SKI. This confirms the critical role of the miR-3180-3p/SKI axis in MASLD pathogenesis. Collectively, these findings highlight miR-3180-3p as both a diagnostic biomarker and a therapeutic target for MASLD, offering new avenues for non-invasive diagnosis and targeted intervention strategies aimed at halting or reversing disease progression.

## Figures and Tables

**Table 1.** Comparison of Clinical Data Between the Normal Group and the NASLD Group

Statistical Indicators	Normal Group (n = 100)	NAFLD Group (n = 117)	Statistic	*P*
Age	49.80 ± 10.50	50.10 ± 11.09	t = 0.205	.840
Gender (male/female)	57/43	64/53	χ^2^ = 0.116	.730
BMI (kg/m^2^)	24.81 ± 2.52	25.08 ± 2.86	t = 0.750	.450
TC (mmol/L)	4.65 ± 0.59	4.78 ± 0.73	t = 1.482	.140
TG (mmol/L)	1.48 ± 0.30	2.15 ± 0.63	t = 9.732	<.001
HDL-C (mmol/L)	1.46 ± 0.18	1.37 ± 0.20	t = 3.212	.002
LDL-C (mmol/L)	2.64 ± 0.41	2.95 ± 0.40	t = 5.654	<.001
WBC (×10^9^/L)	6.07 ± 1.20	6.99 ± 1.60	t = 4.748	<.001
CRP (mg/L)	1.98 ± 0.62	4.14 ± 1.40	t =14.280	<.001
AST (U/L)	21.58 ± 4.97	40.13 ± 12.52	t = 13.910	<.001
ALT (U/L)	22.53 ± 4.12	49.97 ± 13.44	t = 19.630	<.001
DM (No/Yes)	54/46	52/65	χ^2^ = 1.970	.160
EH (No/Yes)	59/41	65/52	χ^2^ = 0.261	.610

ALT, alanine aminotransferase; AST, aspartate aminotransferase; BMI, body mass index; CRP, C-reactive protein; DM, diabetes mellitus; EH, essential hypertension; HDL-C, high-density lipoprotein cholesterol; LDL-C, low-density lipoprotein cholesterol; TC, total cholesterol; TG, triglycerides; WBC, white blood cell.

**Table 2.** Pearson Correlation Analysis was Performed to Investigate the Correlations Between miR-3180-3p and Various Clinical Indicators in Patients

Indicators	Pearson Correlation Coefficient	*P*
TG	0.576	<.01**
HDL-C	−0.619	<.01**
LDL-C	0.539	<.01**
WBC	0.557	<.01**
CRP	0.642	<.01**
OA	0.533	<.01**
PA	0.717	<.01**
FIB-4	0.739	<.001***

CRP, C-reactive protein; FIB-4, Fibrosis-4 Index; HDL-C, high-density lipoprotein cholesterol; LDL-C, low-density lipoprotein cholesterol; OA, oleic acid; PA, palmitic acid; TG, triglycerides; WBC, white blood cell.

## Data Availability

The data that support the findings of this study are available on request from the corresponding author.
